# The Effect of Healthy Lifestyle Promotion Intervention on Quality of Life in Cyclic Mastalgia via Individual Counseling: A Randomized Controlled Clinical Trial

**DOI:** 10.30476/ijcbnm.2020.85560.1274

**Published:** 2021-01

**Authors:** Solmaz Fakhravar, Nasim Bahrami, Mostafa Qurbani, Forouzan Olfati

**Affiliations:** 1 Student Research Committee, School of Nursing and Midwifery, Qazvin University of Medical Sciences, Qazvin, Iran; 2 Social Determinants of Health Research Center, School of Nursing and Midwifery, Qazvin University of Medical Sciences, Qazvin, Iran; 3 Department of Epidemiology, School of Medicine, Alborz University of Medical Sciences, Karaj, Iran; 4 Metabolic Diseases Research Center, School of Nursing and Midwifery, Qazvin University of Medical Sciences, Qazvin, Iran

**Keywords:** Breast Pain, Cyclic, Lifestyle, Quality of life

## Abstract

**Background:**

Despite high number of visited cases, there are no certain therapeutic guidelines for mastalgia. Generally pain is associated with poor quality of life in all dimensions. The present study aimed to investigate the effect of healthy lifestyle promotion intervention on the quality of life in cyclic mastalgia.

**Methods:**

This study was a randomized controlled trial (RCT) conducted on women suffering from cyclic mastalgia at the age of 20 and older before menopausal age referred to the health centers of Karaj,Iran from September 2017 to August 2018. The participants were randomly assigned to intervention (N=40) and control groups (N=40). Intervention was carried out in two 46-minute sessions and two 90-minute sessions for the intervention group. The quality of life was assessed before and after the intervention in both groups using the World Health Organization Quality of Life-BREF (WHOQOL-BREF). The data were analyzed through SPSS statistical software(version 21) using independent sample t-test, Chi-square,and Paired t-test. Besides, P<0.05 was considered as statistically significant.

**Results:**

Before the intervention, both groups were matched in terms of marital status, educational level, occupation, history of breastfeeding, and mean scores of quality of life (P>0.05). After the intervention, the mean score of life quality in physical, social, environmental, and general health dimensions in the intervention group increased significantly compared to the control group (P&gt;0.001). This difference was not statistically significant in the mental health dimension (P=0.086).

**Conclusion:**

The present study indicated that healthy lifestyle promotion intervention could improve the quality of life of women with cyclic mastalgia.

**Trial Registration Number:** IRCT2017100236513N1.

## INTRODUCTION

According to the World Health Organization (WHO), quality of life refers to individuals’ perception of their positions in life in terms of culture; the value system in which they live; and their goals, expectations, standards and priorities. ^1^ Previous studies have found that pain in general could be associated with poor quality of life in all dimensions (physical, mental, and social). They have also shown that reducing pain intensity could improve the quality of life ^2 , 3^ and mastalgia is among the mentioned pains. Mastalgia can be classified into three major categories: cyclic, noncyclical, and extra-mammary. Cyclicmastalgia is thought to be related to hormonal changes. It constitutes two-thirds of female breast pain patients, presenting most severely a week before the onset of their menstrual cycle. This corresponds with the luteal phase and often improves with the onset of menses. It is relieved during menstruation and occurs commonly during the third and fourth decades of life. ^4 , 5^

Mastalgiais a common concern among women, forcing them to regularly refer to healthcare centers for counseling, ^6^ and approximately 70% of women suffer from this pain during their lifetime. ^7^ A study in Hamedan, Iran, indicated that 60% of women suffered from cyclic mastalgia. ^8^ The cause of mastalgia is still unknown. Various studies have indicated that it may result from such factors as an imbalance between estrogen and progesterone hormones, increased levels of prolactin hormone ^9^ and thyroid hormone, ^10^ lack of certain vitamins, ^6^ fat metabolism, ^11^ history of premenstrual syndrome, anxiety, stress, and depression. ^12^

Mastalgia could impair sexual, physical, social, occupational, and educational activities. ^13^ Cyclicmastalgia is responsible for a large number of mammography in young women. Although being largely ignored both scientifically and clinically, this condition merits further biopsychosocial investigation. ^8^ Medical treatments used to treat mastalgia often include chemical treatments with many side effects. These kinds of treatments are often likely to fail. ^14^ Up to 85% of women with breast pain will show alleviation of pain episodes after getting reassurance of not having breast cancer. The remaining 15% will require treatment apart from reassurance, mainly because of the negative impact on physical activity (30%), sexual activity (up to 40%) as well as their life quality in work and social activities (10%). The first-line pain-relieving therapy should be conservative with a trial of at least six months before moving to second-line therapy. ^4^ Counseling services are likely to increase the pain tolerance threshold by reducing fear, anxiety, and concern, creating self-confidence and promoting the understanding of the disease. ^15^ Training-based counseling can be an effective non-drug method to improve life quality and reduce pain by modifying the patients’ life style, i.e. by both improving their nutrition and activities as well as helping them control their weight, stress, and anxiety. First-line management of breast pain should be explanation and, reassurance. ^15 , 16^

Few studies have been conducted on training-based counseling by modifying the patients’ lifestyle in cyclic mastalgia. ^16^ Therefore, the present study aimed to investigate the effect of individual lifestyle promotion-based counseling as a non-drug intervention on the quality of life of women with cyclic mastalgia.

## MATERIALS AND METHODS

The present study was a randomized controlled trial (parallel design). The statistical population consisted of all women referred to five health centers of Karaj city in Iran. Women who met the inclusion criteria were included in the study. Besides, this study was conducted from September 2017 to August 2018. The inclusion criteria were women with mastalgia criterion equal or greater than four based on the pain scale, women whose pain lasts for more than five days per month based on Cardiff’s breast pain score, ^13^ women aged from 20 years old to the menopause age, and those with regular menstruation periods. The exclusion criteria included pregnant women, women with physical and mental illnesses (self-reported), women with a history of breast cancer either individually or related to family members, patients with treated mastalgia in the last three months,and women who used hormone methods of contraception.

By considering α=0.05, β=0.2, and d=8, the sample size was obtained under 35 for each group; however, it reached 40 by considering at least 10% sample drop for each group. ^16^ The sample size was also calculated based on the life quality outcomes.

N=(1.96 + 0.84)2(9.18 2 +13.98 2 )82=35

A random two-stage cluster sampling method was used to select the health centers, where the sampling was carried out. First, a list of health centers of Alborz University of Medical Sciences was prepared. Then, Karaj city was divided into five regions, including northwest, northeast, southwest, southeast, and center. After that, a health center was randomly selected from each region.

After referring to sampling centers, eligible individuals were selected from these centers using available sampling methods. After explaining the aims of the study to the participants, they filled out the Personal-social information questionnaire. The visual scale of pain and Cardiff’smastalgia table were then completed at home by the patients during 2 months. Among them, those with pain intensity equal to or more than 4 and pain duration more than 5 days per month were included in the research. 

The study sample included 80 eligible women with cyclic mastalgia. They were then randomly selected from the health centers (16 individuals from each center). The simple random allocation method was also used to assign women with cyclic mastalgia to either the intervention or control groups. In other words, in each health center, the two groups were previously called A and B. These letters were written on uniform and folded pieces of paper equal to the number of participants. Patients, who referred to each center chose a letter through a lottery without paper replacement. Then, they were enrolled in either group A or B based on their own random selection. Therefore, the affected women were divided into an intervention group of 40 participants (those receiving the counseling) and a control group of 40 participants (those not receiving the counseling).

**Figure 1:**
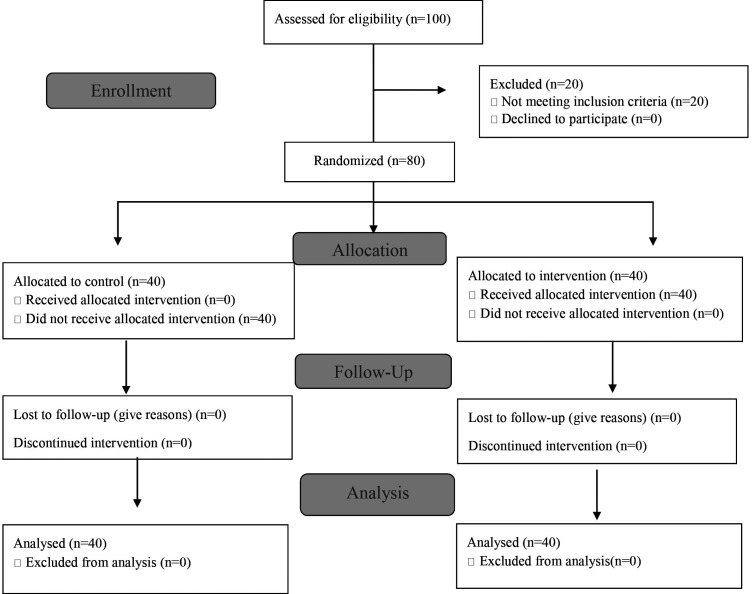
CONSORT flow diagram of design and protocol of the study

The study had as a single-blind design in a way that the participants were not aware of the group they were supposed to be assigned (Figure 1).

**Figure 2:**
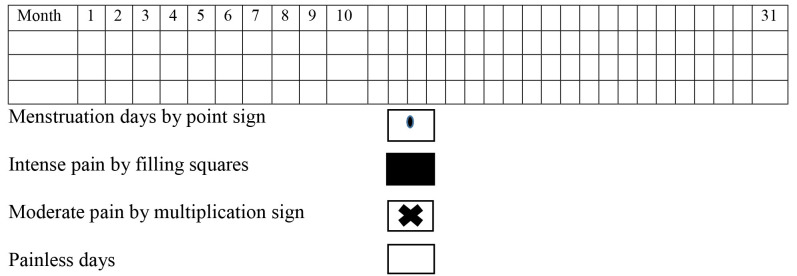
Cardiff’s mastalgia chart

In this study, we also collected the required data through the following instruments;

1. Visual analogue scale (VAS), which is applicable in physiotherapy. We used it to investigate the pain intensity. It is also like a ruler numerated from zero to ten. Accordingly, scores of 1-3, 4-6, 7-9, and 10 represent mild, moderate, severe, and intolerable pain, respectively. The validity and reliability of this tool had been already investigated in a study. ^17^ In this study, the pain intensity scale of 4 and more (moderate and severe) were considered. Due to the high validity and reliability of VAS and the need to use the instrument within a short time, it has been recommended to use it in chronic pains. ^18^

2. Clinic Cardiff’smastalgia chart: It was developed by Clinic Cardiff to determine the duration of pain based on the definition of cyclic mastalgia. ^13 , 19^ As seen, it has 31 squares with scores from 1 to 31, each of which representing the same day of a month (Figure 2).

3. Personal-social information questionnaire: It includes questions about demographic characteristics and history of breastfeeding; history of breast hitting, history of breast surgery or sampling, and history of nipple secretion. The questionnaire was given to five experts of the midwifery department and approved by them in terms of face and content validities.

4. The World Health Organization Quality of Life-BREF (WHOQOL-BREF): This questionnaire assesses four aspects, including physical, psychological, social, and environmental health with 24 questions (7, 6, 3, and 8 questions for each aspect, respectively)and 2 miscellaneous questions surveying the general health condition and life quality level. Each aspect was scored separately (range: 4-20). Score 4 represented the worst, while score 20 showed the best condition. These scores were transformable to the 0-100 scores range. WHOQOL-BREF domain scores demonstrated good discriminant validity, content validity, internal consistency (Cronbach alpha was reported 0.66-0.8) and test–retest reliability. ^20^ Psychometric properties or the validity and reliability of the Persian version of the tool were examined in Iranian community. The reliability of WHOQOL-BREF questionnaire in Iranian community with test-retest of all domains: physical health, mental health, social relations and environmental health respectively 0.77, 0.77, 0.75, 0.84. Also, internal consistency of all domains using alpha Cronbach between patients and healthy subjects was reported 0.52-0.84. The validity of the questionnaire was estimated by known groups’ comparison and convergent validity. Since the WHOQOL-BREF demonstrated a statistically significant correlation with the Iranian version of the SF-36, as it was expected, the convergent validity of the questionnaire was found to be desirable. The correlation matrix also showed satisfactory results in all domains, except for social relationships. Qualitative content validity was also reported optimal. ^21^

Before the intervention, both the intervention and control groups responded to the study questionnaires. The intervention was conducted in the form of individual counseling sessions. The four counseling sessions included two 45-minute sessions and two 90- minute sessions. The first three sessions were held every other day and the last session was held a month after the third counseling session. The two groups also completed the life quality questionnaire one month after the third counseling session in the fourth session.

In counseling sessions, the researcher outlined the subject of the session and invited the patients to enter the discussion after providing information on the subject in a simple language through questioning and survey to identify the patients’ needs and problems. Afterward, necessary explanations on the proper problem-solving solutions were provided with the patients’ active participation and cooperation. After the end of the research, the individual counseling sessions were also held for the control group (those who were willing to) similar to the intervention group to comply with the ethical principles in the research. 

The content of the sessions was based on modifying the participants’ lifestyles.

**Session 1:** Introduction and statement of problem by clients. The counseling goals were introduced to the patients. They also became familiar with breast anatomy, its physiology, sex hormones as well as other hormones and their effect on breast function, the concept of mastalgia and its causes, its effects on various aspects of life, and the effects of various life factors on cyclic mastalgia in a simple language. 

**Session 2:** The patients became acquainted with ways of dealing with cyclic mastalgia, diet, physical activity and its effects on the improvement of this disease’s symptoms.

**Session 3:** The patients were acquainted with stress and its control methods as well as how stress could be related to cyclic mastalgia.In this session, the patient’s questions were also answered.

**Session 4:** The effect of counseling was followed up and the questionnaires were filled out. 

After collection of data, they were analyzed by SPSS 21. In this study, descriptive statistics were used to provide general information; independent t-test and Paired t-test were applied to compare the intervention and control groups before and after the intervention, and the chi-square test was utilized to compare the qualitative variables. The significance level of the tests was less than 0.05.

**Ethical statement:** The study was conducted after obtaining permission from the Ethics Committee of Qazvin University of Medical Sciences (Ethic Committee code: IR.QUMS.REC.1396.246), registered in the Iranian Registry of Clinical Trials (IRCT). We also obtained a letter of recommendation from the Faculty of Nursing and midwifery to be allowed to attend the study centers. Written informed consents were further received from the subjects. 

## RESULTS

There was no significant difference between the two groups in terms of demographic characteristics. The mean age of the intervention group was 37.65±6.61, while it was 36.95±7.50 for the control group (P=0.433). Most of the research participants (73=91.3%) were married. 36 (45%) participants had high school diploma and 54 (67.5%) of them were housewives (Table 1).

**Table 1:** Comparison of the demographic characteristics of the two groups

Variable		Intervention N=40	Control N=40	P value*
	Group	N (%)	N (%)	
Marital status	Single	4 (10%)	3 (7.5%)	0.692
	Married	36 (90%)	37 (92.5%)	
Education level	Illiterate	5 (12.5%)	3 (7.5%)	0.450
	Elementary school degree	9 (22.5%)	5 (12.5%)	
	High school diploma	15 (37.5%)	21 (52.5%)	
	Bachelor	11 (27.5%)	10 (25%)	
	Higher education	-	1 (2.5%)	
Job	Housewife	28 (70%)	26 (65%)	0.885
	Governmental job	4 (10%)	5 (12.5%)	
	Self-employed	8 (20%)	9 (22.5%)	
Lactation history		30 (75%)	32 (80%)	0.592

* Chi-square test

At first, the normality of life quality scores was evaluated by the Kolmogorov-Smirnov and Shapiro tests (P=0.187). The comparison between the two groups’ mean scores of the total life quality before individual counseling indicated that the difference of the scores was not statistically significant (P=0.333). After the intervention, the comparison between the groups’ mean scores of quality of life dimensions indicated that the groups were significantly different (P<0.05). However, after the intervention,the two groups were not statistically different in terms of mental health (P=0.086). Also, in the intervention group, comparing the total scores quality of life before and after the intervention showed a statistically significant difference (P=0.016), but this difference was not significant in the control group (P=0.228) (Table 2).

**Table 2:** Comparison of the quality of life scores before and after the intervention between the two groups

	Group	Intervention Mean±SD	Control Mean±SD	P value**
Variable/time				
Physical health	Before	62.67±14.80	62.41±14.26	0.935
	After	67.32±13.88	60.98±13.61	0.008
	P value*	0.020	0.390	
Mental health	Before	61.66±16.37	58.85±15.45	0.432
	After	65.52±18.60	58.54±14.61	0.086
	P value *	0.179	0.831	
Social health	Before	60.00±19.62	57.62±16.10	0.535
	After	65.62±16.25	55.62±17.84	0.001
	P value *	<0.001	0.323	
Environmental health	Before	65.85±13.65	61.25±12.37	0.118
	After	69.29±11.58	59.37±15.45	0.005
	P value *	0.081	0.197	
General health	Before	64.68±18.75	60.31±17.19	0.280
	After	69.66±16.23	59.68±16.62	0.011
	P value *	0.037	0.793	
Total score of quality of life	Before	63.26±13.14	60.50±12.20	0.333
	After	67.50±10.28	59.20±11.83	<0.001
	P value *	0.016	0.228	

* Paired t-test

** Independent t-test

## DISCUSSION

The present study investigated the impact of individual counseling based on lifestyle modification on the life quality of women with cyclic mastalgia and showed that individual counseling could significantly increase the overall life quality scores in the intervention group compared with the control group. In a study, after a 50-minute mental training session and comparing the total life quality scores of the intervention and control groups, two months after the intervention, the results indicated a significant improvement of life quality in the intervention group; hence, this study showed that mental training could improve the life quality of women with mastalgia. ^16^ However, regarding the mental dimension, the above-mentioned study was not in line with the results of the present study.

Furthermore, the current study results were similar to those of a study, which investigated the impact of yoga therapy on the life quality and depression in nursing students with mastalgia before the menopause age. The obtained results indicated that all aspects of life (mental, physical, emotional, and social) significantly promoted in the intervention group, three and six months after the intervention. This research indicated that yoga therapy could improve the quality of life in women with mastalgia. ^22^ The consistency of the above-mentioned study with the present one may be related to an individual counseling session about stress management and implementation of relaxation commands for female patients. Relaxation has been found to be highly effective as a therapeutic strategy for painful and stressful situations. ^23^

Similarly, the present study was inconsistent with the result of a study on the effect of acupuncture on 22 women with mastalgia. The results indicated that there was no significant improvement in all dimensions of life quality (mental, physical, emotional, and social), but a significant reduction was observed in the women’s pain scores. ^24^ Acupuncture, an aggressive therapeutic intervention, seemed to be unable to affect the patients’ quality of life. Since counseling is a process that simultaneously examines and involves all aspects of life in patients, it can be concluded that this intervention has a better impact on individuals’ life quality than acupuncture aiming to reduce pain.

On the other hand, in the present study, despite the higher scores of mental dimension at the end of the study compared to the beginning of the study in the intervention group, this increase was not significant probably due to lack of specific techniques, such as cognitive-behavioral therapy and mindfulness. There may be also a relationship between mastalgia and depression in young women with mastalgia; however, a closer relationship between anxiety and mastalgia has been observed. ^3^

In the present study, lifestyle modification, such as making changes to the daily diet by reducing caffeine intake; reducing saturated fat, salt and simple sugars; exercising;and reassuring the patient that mastalgiais non-cancerous, as an essential part of counseling sessions, seemed to have positive effects on reduction of mastalgia intensity, and subsequently improvement of the individuals’ life quality. Low-fat diet has also shown reducing effect on mastalgia by balancing the amounts of steroid hormones and Gonadotropin. ^13^ Caffeine-free diet could further improve mastalgia through reduction of circular nucleotides followed by a decrease in Kinase protein. ^25^ Also, aerobic exercise has been proved to be efficient in decreasing mastalgia through increasing serum beta-endorphin levels, immune response of the body, quality of sleep, and individual satisfaction followed by a reduction in pain intensity. ^26^

Therefore, first-line management of breast pain should be explanation, reassurance, and a bra-fitting advice. Rassurance provides the patients with mental balance and subsequently increases their pain tolerance to assure them and make them not be frightened of cancer. ^15^

In the current work, at the beginning of the counseling session, after a detailed clinical examination of patients, there was an attempt to reassure them of the non-cancerous nature of mastalgia and explain the possible causes of this pain. Moreover, the counseling sessions played an effective role in reducing pain and increasing the individual’s ability to make appropriate health decisions and learn skills and attitudes to promote self-care for mastalgia. This increase in the individual understanding will lead to improved lifestyles and appropriate health behaviors.

The strength of this study was that it considered the first-line intervention for the treatment of this idiopathic disease (Cyclic mastalgia). On the other side, the limitation of this study was the absence of the second and third types of blinding in the study design because of the type of intervention, which was individual counseling.

## CONCLUSION

The present study indicated that counseling with an emphasis on changing lifestyle could improve the life quality of women with cyclic mastalgia. Lifestyle-based counseling can be one of the first-line treatments for this disease. Thus, because of the high incidence of mastalgia and the efficacy and availability of individual counseling, this method is suggested for women with mild-to-moderate pain. However,further studies are recommended using special psychotherapy techniques.
